# Soluble Receptor for Advanced Glycation End-Products (sRAGE), Glycemic, and Lipid Indices in Diabetic Patients With and Without Microvascular Complications: A Cross-Sectional Study

**DOI:** 10.7759/cureus.117370

**Published:** 2026-10-02

**Authors:** Sweta Singh, Rajlaxmi Sarangi, Suman Tripathy, Tapaswini Pradhan, Krishna P Tripathy, Sambit Das, Shrutakirty Parida, A. Raj Kumar Patro, Jyoti Prakash Sahoo

**Affiliations:** 1 Biochemistry, Kalinga Institute of Medical Sciences, Bhubaneswar, IND; 2 General Medicine, Kalinga Institute of Medical Sciences, Bhubaneswar, IND; 3 Endocrinology, Kalinga Institute of Medical Sciences, Bhubaneswar, IND; 4 Ophthalmology, Kalinga Institute of Medical Sciences, Bhubaneswar, IND; 5 Microbiology, Kalinga Institute of Medical Sciences, Bhubaneswar, IND; 6 Pharmacology, Kalinga Institute of Medical Sciences, Bhubaneswar, IND

**Keywords:** advanced glycation end-products, age-rage signaling pathway, diabetic autonomic neuropathy, diabetic microvascular complications, diabetic nephropathy (dn), diabetic retinopathy, homeostatic model assessment of insulin resistance (homa-ir), serum fasting insulin, soluble rage, triglyceride-glucose index (tyg)

## Abstract

Background and objective

Microvascular complications and insulin resistance in patients with type 2 diabetes mellitus (T2DM) increase the healthcare burden. The homeostatic model assessment of insulin resistance (HOMA-IR) and fasting insulin concentrations are widely used to assess insulin resistance. In resource-limited settings, the triglyceride-glucose (TyG) index is preferred as an alternative measure of insulin resistance. There is debate about the impact of soluble receptor for advanced glycation end-products (sRAGE) on microvascular complications and insulin resistance. However, Indian studies exploring this association are scarce. Therefore, we planned to evaluate sRAGE levels, fasting serum insulin, HOMA-IR, and the TyG index in diabetic individuals with and without microvascular complications. We also analyzed the correlation between sRAGE and other parameters.

Methods

This cross-sectional study was conducted from July 2024 to June 2026 at Kalinga Institute of Medical Sciences, Bhubaneswar, India. Adults with T2DM were enrolled in this study. Those participating in any other research projects were excluded. The study participants were grouped according to the number of microvascular complications (i.e., 0, 1, 2, or 3). We evaluated and compared the following parameters among participants in the four groups: age, duration of T2DM, BMI, fasting blood sugar (FBS), glycated hemoglobin (HbA1c), serum creatinine, estimated glomerular filtration rate (eGFR), serum insulin, serum triglycerides, serum total cholesterol, and sRAGE levels. HOMA-IR and the TyG index were calculated for the participants. We also assessed correlations between sRAGE levels and the collected parameters. We used R software (version 4.6.2; R Foundation for Statistical Computing, Vienna, Austria) for data analysis.

Results

Our study population consisted of 151 patients with diabetes (median age, 54.0 (46.0-63.5) years; 86 males (56.95%)), with a median T2DM duration of 12 years and a median BMI of 27.11 (25.52-28.90) kg/m². Of the 151 participants, 57 (37.75%) had one of the three microvascular complications, 33 (21.85%) had two complications, and 23 (15.23%) had three complications. Thirty-eight participants (25.17%) had no microvascular complications. The median serum creatinine and eGFR levels were 0.91 (0.81-1.96) mg/dL and 71.76 (29.09-95.03) mL/min/1.73 m², respectively. The median FBS, HbA1c, and fasting insulin levels were 189.0 (163.5-217.0) mg/dL, 7.31 (7.03-7.89%), and 4.91 (4.04-6.03) µIU/mL, respectively. The median serum total cholesterol and triglyceride levels were 178.0 (153.0-203.0) mg/dL and 136.0 (116.0-161.5) mg/dL, respectively. The median sRAGE level in the study population was 276.4 (201.8-413.9) pg/mL. The median HOMA-IR and TyG index values were 2.18 (1.78-3.03) and 9.44 (9.22-9.71), respectively. There were negative correlations between sRAGE and serum creatinine (r = -0.561, p < 0.001) and the TyG index (r = -0.332, p < 0.001). The correlation between sRAGE and serum insulin (r = 0.220, p = 0.007) was weakly positive. The correlation between sRAGE and HOMA-IR (r = -0.002, p = 0.976) was not statistically significant.

Conclusions

We found reduced sRAGE levels among participants with microvascular complications. The TyG index and HOMA-IR values were higher among participants with two to three complications. We did not observe strong correlations between sRAGE and any of the studied parameters.

## Introduction

Type 2 diabetes mellitus (T2DM) is one of the most common chronic metabolic disorders worldwide and represents a major public health challenge because of its microvascular complications (i.e., diabetic neuropathy, retinopathy, and nephropathy) [1,2]. In India, there were 264 individuals with T2DM per 1 lakh population in 2021 [3]. The rising incidence of T2DM in India over the last three decades is a public health concern [3]. T2DM continues to be a key contributor to chronic kidney disease, peripheral neuropathy, preventable blindness, lower limb amputation, and early cardiovascular issues, even with significant advancements in diagnosis and treatment [1-4]. These issues considerably lower the quality of life and raise healthcare costs, especially in low- and middle-income countries (LMICs) [2,4]. They also place a substantial burden on India’s healthcare system by increasing morbidity and mortality due to T2DM [3]. Through processes including oxidative stress, inflammation, the polyol and hexosamine pathways, protein kinase C activation, mitochondrial damage, endothelial dysfunction, and the formation of advanced glycation end-products (AGEs), persistent hyperglycemia causes tissue damage [5,6]. AGEs interact with AGE receptors (RAGE) [5]. Among the aforementioned pathways, the AGE-RAGE signaling cascade represents a key molecular interaction linking microvascular complications, microangiopathy, and hyperglycemia [5,6].

Proteins, lipids, and nucleic acids undergo oxidative alteration and nonenzymatic glycation during chronic hyperglycemia, resulting in the production of AGEs [5,7]. AGEs progressively accumulate in the blood and other tissues, such as vascular smooth muscle cells, endothelial cells, macrophages, monocytes, podocytes, Schwann cells, neurons, and retinal pigment epithelial cells [5-7]. Numerous cellular signaling pathways, including the nuclear factor kappa-B (NF-κB) and mitogen-activated protein kinase pathways, are triggered upon AGE-RAGE interactions [5,7,8]. Oxidative stress, inflammation, proinflammatory cytokine release, endothelial dysfunction, extracellular matrix deposition, fibrosis, and apoptosis are all driven by the activation of these signaling networks [6-8]. Furthermore, activation of the NF-κB pathway increases RAGE expression, triggering a positive feedback loop that leads to persistent inflammation [6,7,9].

Soluble RAGE (sRAGE) is a variant of RAGE that prevents the hyperactive response of the AGE-RAGE axis [5,10]. The extracellular domain of sRAGE is responsible for blocking AGE-RAGE signaling [10,11]. sRAGE is produced by proteolytic cleavage of membrane-bound RAGE by matrix metalloproteinase-9 and by splicing of the AGER gene [10-12]. Some studies have found decreased serum sRAGE concentrations in patients with diabetes and microvascular complications [13,14]. Conversely, other studies have reported increased sRAGE concentrations in patients with T2DM, which may be explained by elevated inflammation, receptor shedding, and reduced renal clearance [15,16]. These discrepancies might be attributed to genetic variations in the AGER gene, ethnicity, duration of diabetes, glycemic control, renal function, and ongoing medications [13,15,17].

Insulin resistance plays a significant role in the pathogenesis of dysglycemia and microvascular complications [18]. Insulin resistance leads to impaired glucose utilization by peripheral tissues, increased hepatic gluconeogenesis, dyslipidemia (hypercholesterolemia and hypertriglyceridemia), vascular dysfunction, oxidative stress, and inflammation [18,19]. Fasting serum insulin and the homeostatic model assessment of insulin resistance (HOMA-IR) score are reliable surrogate markers of insulin resistance [18,19]. Recent studies have revealed strong bidirectional correlations between insulin resistance and the AGE-RAGE axis [9]. Activation of the RAGE axis leads to insulin resistance by enhancing the phosphorylation of insulin receptor substrates and activating inflammatory pathways [18]. Insulin resistance and hyperinsulinemia lead to oxidative stress and accelerated AGE production [5]. The triglyceride-glucose (TyG) index can also be considered a surrogate biomarker for insulin resistance in LMICs, where serum insulin estimation is not feasible [20]. Measurement of sRAGE, HOMA-IR, and the TyG index could therefore be helpful for assessing insulin resistance in patients with diabetes, with and without complications.

There are limited data on the interactions among serum sRAGE, fasting insulin, HOMA-IR, and the TyG index in Indian patients with diabetes, with or without microvascular complications. Therefore, the present study was undertaken to compare serum sRAGE levels, fasting serum insulin concentrations, HOMA-IR, and the TyG index among patients with diabetes, with and without microvascular complications. We also evaluated the glycemic, lipid, and renal parameters of the participants. The findings might improve biomarker-based risk stratification, provide greater insight into the interaction between insulin resistance and the AGE-RAGE axis, and clarify the potential clinical utility of sRAGE as an adjunctive biomarker for identifying patients at increased risk of diabetic microvascular complications.

## Materials and methods

Study design and ethical approval

This cross-sectional study was conducted between July 2024 and June 2026. We obtained ethical approval (KIIT/KIMS/IEC/1858/2024, dated June 25, 2024) from the Institutional Ethics Committee before commencing the study. Written informed consent was obtained from each participant before enrollment.

Study participants

Adult female and male individuals with T2DM were included in this study. Patients with hypertension, thyroid disorders, or any malignancy were excluded. Pregnant women and nursing mothers were also excluded. Patients enrolled in other studies were excluded as well.

Study objectives

We planned this study to measure sRAGE levels and AGER gene polymorphisms in patients with diabetes, with and without microvascular complications. This paper presents the first part of the larger study. The study participants were categorized according to the number of microvascular complications (i.e., 0, 1, 2, or 3). We evaluated and compared the following parameters among participants in the four groups: age, BMI, duration of T2DM, fasting blood sugar (FBS), glycated hemoglobin (HbA1c), serum creatinine, estimated glomerular filtration rate (eGFR), serum insulin, serum total cholesterol, serum triglycerides, and sRAGE levels. We also calculated the HOMA-IR and TyG index values and compared them across the four groups. Correlating sRAGE levels with these parameters was an additional objective. The second paper will focus on the association between AGER gene polymorphisms and insulin resistance in patients with diabetes, with and without microvascular complications.

Study procedure

Under strict aseptic conditions, an 8-mL blood sample was collected from the median cubital vein of each participant after an overnight fast of eight to 12 hours. Of this sample, 2 mL was collected in a fluoride vial to measure FBS. For HbA1c estimation, 3 mL of blood was collected in an ethylenediaminetetraacetic acid vial. HbA1c estimation was performed by high-performance liquid chromatography using a Tosoh HLC-723GX® analyzer (Tosoh Corporation, Tokyo, Japan). The remaining 3 mL of venous blood was collected in a red-top vacutainer. After centrifugation at 2,000-3,000 rpm for 10 minutes, 1 mL of serum was removed, immediately aliquoted, and stored at -70°C for sRAGE analysis. The remaining serum was used to analyze insulin, lipid profile, and creatinine. Serum creatinine was quantified using a DxC 700 AU Beckman Coulter autoanalyzer® (Beckman Coulter, Inc., Brea, CA, USA).

sRAGE was quantified using a human sRAGE enzyme-linked immunosorbent assay (ELISA) kit (Lablisa, LabReCon, New Delhi, India; catalog number LAB8458) according to the manufacturer’s protocol. The intra-assay and inter-assay coefficients of variation for the sRAGE ELISA kit were less than 8% and less than 10%, respectively. The analytical measurement range for sRAGE was 12.5-800 pg/mL. The sandwich immunoassay was performed according to the kit instructions. Purified human sRAGE antibody was coated onto the microtiter plate, forming a solid-phase antibody layer. When specimens were added to the wells, sRAGE in the specimens bound to the antibody and a biotin-conjugated antibody specific to human sRAGE. Avidin conjugated to horseradish peroxidase was then added to each microplate well and incubated to form an antibody-antigen-enzyme-antibody complex. After washing, a tetramethylbenzidine substrate was added, resulting in a color change. The reaction was then terminated by adding sulfuric acid as the stop solution. The color change was measured spectrophotometrically at 450 nm using a PR4100 ELISA reader (Bio-Rad, Hercules, CA, USA). The concentration of sRAGE was determined by comparing the specimen’s optical density with the standard curve.

The Access ultrasensitive insulin assay (Beckman Coulter, Inc.) is a sandwich immunoassay that uses paramagnetic particles and chemiluminescence to quantitatively determine insulin levels in human serum. The sample was added to a reaction vessel along with mouse monoclonal anti-insulin alkaline phosphatase conjugate and paramagnetic particles coated with mouse monoclonal anti-insulin antibody. After incubation, the materials bound to the solid phase were held in a magnetic field while the unbound materials were washed away. The chemiluminescent substrate was added to the vessel, and the light generated by the reaction was measured with a luminometer. The amount of analyte in the sample was determined using a stored, multipoint calibration curve. The normal range of serum insulin is 1.9-23 µIU/mL.

The eGFR was calculated using the Modification of Diet in Renal Disease (MDRD) formula [21]. According to the MDRD formula, eGFR is calculated as follows:

$\mathrm{eGFR} = 175 \times \mathrm{SCr}^{-1.154} \times \mathrm{Age}^{-0.203} \times 0.742 \text{ (if female)} \times 1.21 \text{ (if Black)}$

Based on eGFR values, kidney disease is classified into the following stages: Stage I, eGFR ≥ 90 mL/min/1.73 m²; Stage II, eGFR 60-89 mL/min/1.73 m²; Stage III, eGFR 30-59 mL/min/1.73 m²; Stage IV, eGFR 15-29 mL/min/1.73 m²; and Stage V, eGFR < 15 mL/min/1.73 m².

The HOMA-IR was calculated using the following formula [22]:

$\mathrm{HOMA-IR} = \frac{\text{Fasting insulin }(\mu\mathrm{IU/mL}) \times \text{Fasting glucose }(\mathrm{mg/dL})}{405}$

Its normal range is 0.7-2.0. Higher HOMA-IR values (>2.0) indicate increased insulin resistance [22]. The TyG index was calculated using the following formula [23]:

$\text{TyG index} = \ln\left(\frac{\text{Fasting triglycerides (mg/dL)} \times \text{Fasting glucose (mg/dL)}}{2}\right)$

The TyG index is measured on a logarithmic scale. Scores below 8.5 indicate good insulin sensitivity and lower metabolic risk, whereas scores above 9.0 indicate reduced insulin sensitivity and an increased risk of cardiovascular issues [23].

Statistical analysis

Our goal was to investigate the relationships between sRAGE levels and AGER gene polymorphisms in patients with diabetes, with and without microvascular complications. Considering the pilot nature of this study and the cost of assessing gene polymorphisms, we planned to enroll approximately 150 participants. Therefore, we employed nonprobability convenience sampling.

We assessed the normality of the data distribution using the Kolmogorov-Smirnov test. The results indicated that the collected data were nonparametric. Continuous and categorical data were presented as medians with IQRs and frequencies with proportions, respectively. The Kruskal-Wallis test was used to analyze quantitative data. The Bonferroni test was used for post hoc analysis. Categorical data were analyzed using the chi-square test. Spearman’s rank correlation was used for correlation analysis. We used R software (version 4.6.2; R Foundation for Statistical Computing, Vienna, Austria) [24] for data analysis and plot generation. All statistical tests were two-tailed. Statistical significance was defined as p < 0.05.

## Results

We conducted this cross-sectional study from July 2024 to June 2026. We screened 2,073 patients for this study. Of these, 1,684 (81.24%) had already been enrolled in other studies, and 238 (11.48%) declined to participate. The remaining 151 patients (7.28%) were enrolled in this study. They were divided into four study groups based on the number of microvascular complications. Table 1 shows their clinical and demographic parameters. Of the 151 participants, 57 (37.75%) had one complication, 33 (21.85%) had two complications, and 23 (15.23%) had three complications. Thirty-eight participants (25.17%) had no microvascular complications. The median age of the study population was 54.0 (46.0-63.5) years. Eighty-six males comprised 56.95% of the study population. The median duration of T2DM was 12.0 (8.0-16.0) years. The median BMI was 27.11 (25.52-28.90) kg/m². The median eGFR was 71.76 (29.09-95.03) mL/min/1.73 m². Between-group assessments revealed statistically significant differences in all parameters except sex. These findings may be associated with the number of microvascular complications among the participants.

**Table 1:** Demographic and clinical parameters of the study participants. Continuous data are expressed as medians and IQRs. Categorical data are expressed as frequencies and proportions. Continuous and categorical data were assessed using the Kruskal-Wallis test and the chi-square (χ²) test, respectively. The H-statistics, chi-square statistics, and p-values were derived. A p-value < 0.05 was considered statistically significant. eGFR: estimated glomerular filtration rate; T2DM: type 2 diabetes mellitus

Parameters	Total participants (n = 151)	Participants without any complication (n = 38)	Participants with one complication (n = 57)	Participants with two complications (n = 33)	Participants with three complications (n = 23)	Statistical test used	Test statistic	p-value
Age (years)	54.0 (46.0-63.5)	43.0 (39.3-47.0)	57.0 (48.0-64.0)	61.0 (53.0-68.0)	61.0 (55.5-67.0)	Kruskal-Wallis test	64.28	<0.001
Age >65 years	28 (18.54%)	0	9 (15.79%)	12 (36.36%)	7 (30.43%)	Chi-square test	18.03	<0.001
Male sex	86 (56.95%)	24 (63.16%)	32 (56.14%)	17 (51.51%)	13 (56.52%)	Chi-square test	1.01	0.798
BMI (kg/m²)	27.11 (25.52-28.90)	28.80 (28.05-30.07)	27.19 (25.70-29.46)	26.03 (24.81-27.35)	25.35 (24.48-26.26)	Kruskal-Wallis test	52.08	<0.001
Duration of T2DM (years)	12.0 (8.0-16.0)	7.0 (4.0-8.8)	12.0 (9.0-15.0)	16.0 (11.0-21.0)	15.0 (12.0-19.5)	Kruskal-Wallis test	63.07	<0.001
Serum creatinine (mg/dL)	0.91 (0.81-1.96)	0.81 (0.67-0.87)	0.87 (0.81-1.58)	1.93 (1.06-2.13)	2.60 (2.38-2.82)	Kruskal-Wallis test	86.82	<0.001
eGFR (mL/min/1.73 m²)	71.76 (29.09-95.03)	98.62 (93.29-109.47)	83.39 (43.22-94.29)	30.87 (26.48-70.56)	22.65 (19.48-25.02)	Kruskal-Wallis test	100.91	<0.001

Figure 1 illustrates the distribution of the study participants using a Voronoi treemap. The four colors (i.e., green, yellow, blue, and red) denote the number of microvascular complications (i.e., 0, 1, 2, or 3) among participants. The sizes of the large and small blocks depend on the number of participants they represent. Each entry in the plot has five alphanumeric characters. The first and second characters indicate the age group (A: adult; E: elderly) and sex (M: male; F: female), respectively. The third and fourth characters indicate the duration of T2DM (N: <10 years; S: 10-20 years; L: >20 years) and glycemic control, as indicated by HbA1c (C: <7.0%; U: 7.0-8.0%; P: >8.0%), respectively. The fifth character indicates the number of microvascular complications (i.e., 0, 1, 2, or 3). Twelve (7.95%) adult male participants with T2DM for <10 years and an HbA1c of 7.0-8.0% had no complications (shown as AMNU0 in the plot). Eleven (7.28%) adult female participants with T2DM for 10-20 years and an HbA1c of 7.0-8.0% had one microvascular complication (shown as AFSU1 in the plot). Nine (5.96%) adult male participants with T2DM for 10-20 years and an HbA1c of 7.0-8.0% had one microvascular complication (shown as AMSU1 in the plot). Eight (5.30%) adult female participants with T2DM for 10-20 years and an HbA1c of 7.0-8.0% had two microvascular complications (shown as AFSU2 in the plot). Six (3.97%) adult male participants with T2DM for <10 years and an HbA1c <7.0% had no microvascular complications (shown as AMNC0 in the plot). Another six (3.97%) adult male participants with T2DM for <10 years and an HbA1c <7.0% had one complication (shown as AMNC1 in the plot). Six (3.97%) adult male participants with T2DM for 10-20 years and an HbA1c >8.0% had all three microvascular complications (shown as AMSP3 in the plot).

**Figure 1:**
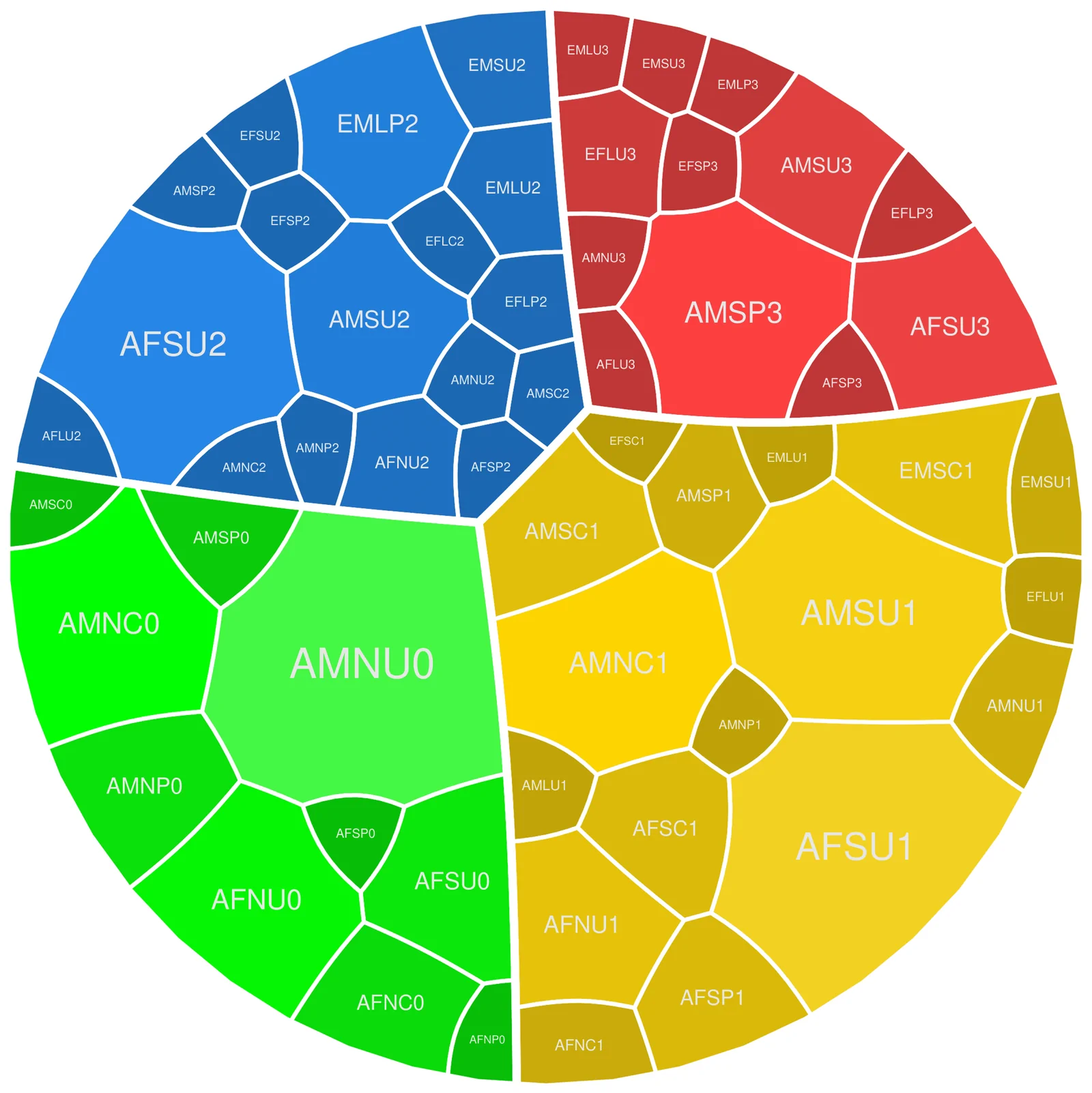
Distribution of the study population. The Voronoi treemap illustrates the distribution of the study population. Each alphanumeric data item has five characters. The first and second characters represent the age group (A: adult; E: elderly) and sex (M: male; F: female), respectively. The third and fourth characters represent the duration of T2DM (N: <10 years; S: 10-20 years; L: >20 years) and glycemic control, as indicated by HbA1c (C: <7.0%; U: 7.0-8.0%; P: >8.0%), respectively. The final character represents the number of microvascular complications (i.e., 0, 1, 2, or 3). The colors of the Voronoi treemap also reflect the number of microvascular complications among participants. HbA1c: glycated hemoglobin; T2DM: type 2 diabetes mellitus R software (version 4.6.2; R Foundation for Statistical Computing, Vienna, Austria) was used to generate this plot [24].

Age, duration of T2DM, and serum creatinine

Figure 2 illustrates the age distribution of the participants. The median age of the entire study population was 54.0 (46.0-63.5) years. The median ages of participants with 0, 1, 2, and 3 complications were 43.0 (39.3-47.0), 57.0 (48.0-64.0), 61.0 (53.0-68.0), and 61.0 (55.5-67.0) years, respectively (p < 0.001). Younger individuals had fewer complications than older individuals. Approximately one-third of participants with ≥ 2 complications were elderly. The age distribution is also shown in Table 1.

**Figure 2:**
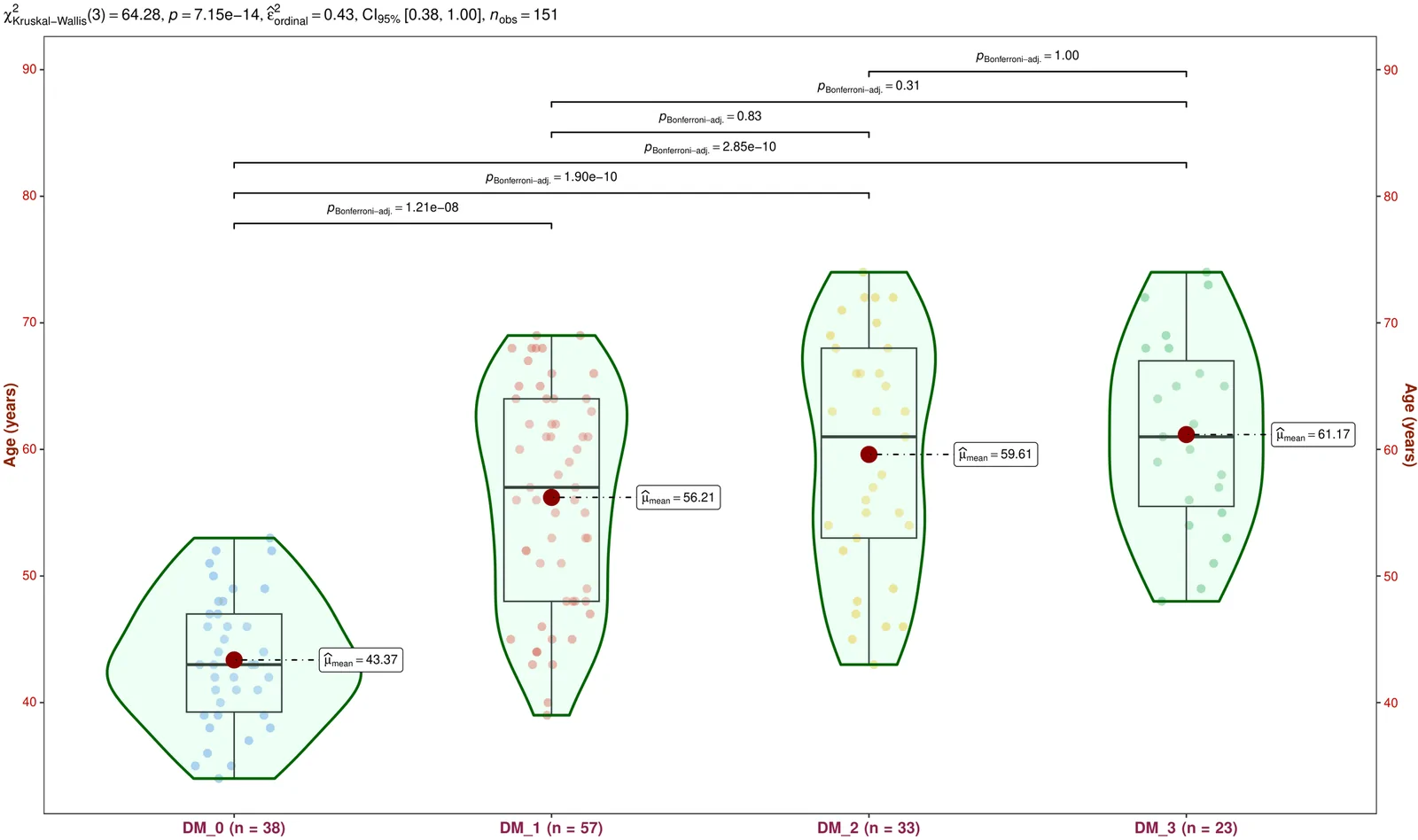
Age distribution of the study population. The violin, jitter, and box-and-whisker plots illustrate the ages of the study participants (n = 151). The red dots denote the mean values. The Kruskal-Wallis test was used to assess these data. For post hoc analysis, we used the Bonferroni test. A p-value < 0.05 was considered statistically significant. The digits (i.e., 0, 1, 2, and 3) in the groups shown on the x-axis represent the number of microvascular complications. T2DM: type 2 diabetes mellitus R software (version 4.6.2) was used to generate this plot [24].

Figure 3 illustrates the duration of T2DM among the participants. The median duration was 12.0 (8.0-16.0) years. The median durations of T2DM among participants with 0, 1, 2, and 3 complications were 7.0 (4.0-8.8), 12.0 (9.0-15.0), 16.0 (11.0-21.0), and 15.0 (12.0-19.5) years, respectively (p < 0.001). Participants with a longer duration of T2DM had more complications. The duration of T2DM is also shown in Table 1.

**Figure 3:**
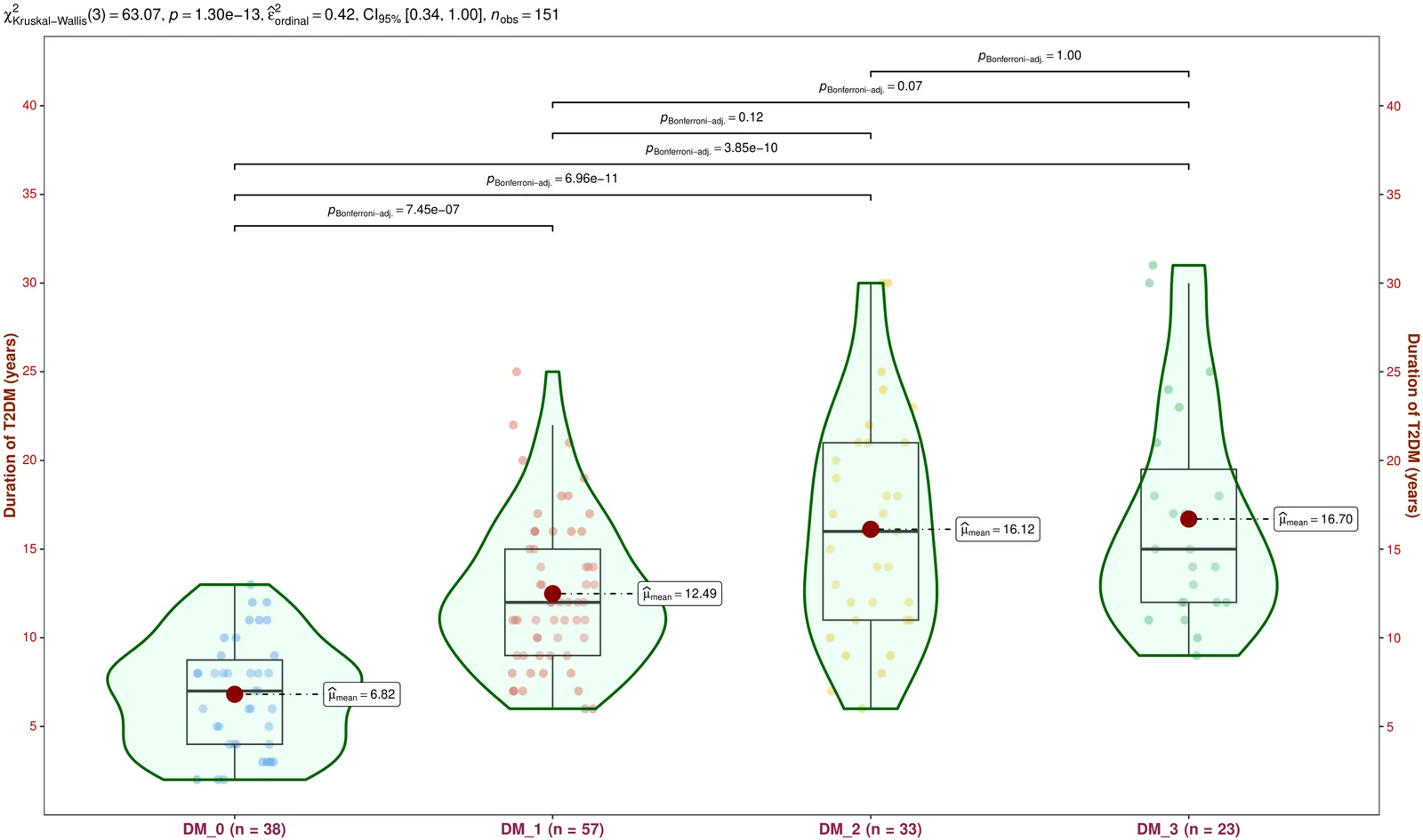
Duration of T2DM of the study participants. The violin, jitter, and box-and-whisker plots illustrate the duration of T2DM among the study participants (n = 151). The red dots denote the mean values. The Kruskal-Wallis test was used to assess these data. For post hoc analysis, we used the Bonferroni test. A p-value < 0.05 was considered statistically significant. The digits (i.e., 0, 1, 2, and 3) in the groups shown on the x-axis represent the number of microvascular complications. T2DM: type 2 diabetes mellitus R software (version 4.6.2) was used to generate this plot [24].

Figure 4 illustrates the serum creatinine levels of the participants. The median serum creatinine level was 0.91 (0.81-1.96) mg/dL. The median serum creatinine levels among participants with 0, 1, 2, and 3 complications were 0.81 (0.67-0.87), 0.87 (0.81-1.58), 1.93 (1.06-2.13), and 2.60 (2.38-2.82) mg/dL, respectively (p < 0.001). The mean serum creatinine levels showed an increasing trend with the number of complications. The bimodal distribution among participants with one to two complications could be attributed to the presence of diabetic nephropathy in some participants. The participants’ serum creatinine levels are also shown in Table 1.

**Figure 4:**
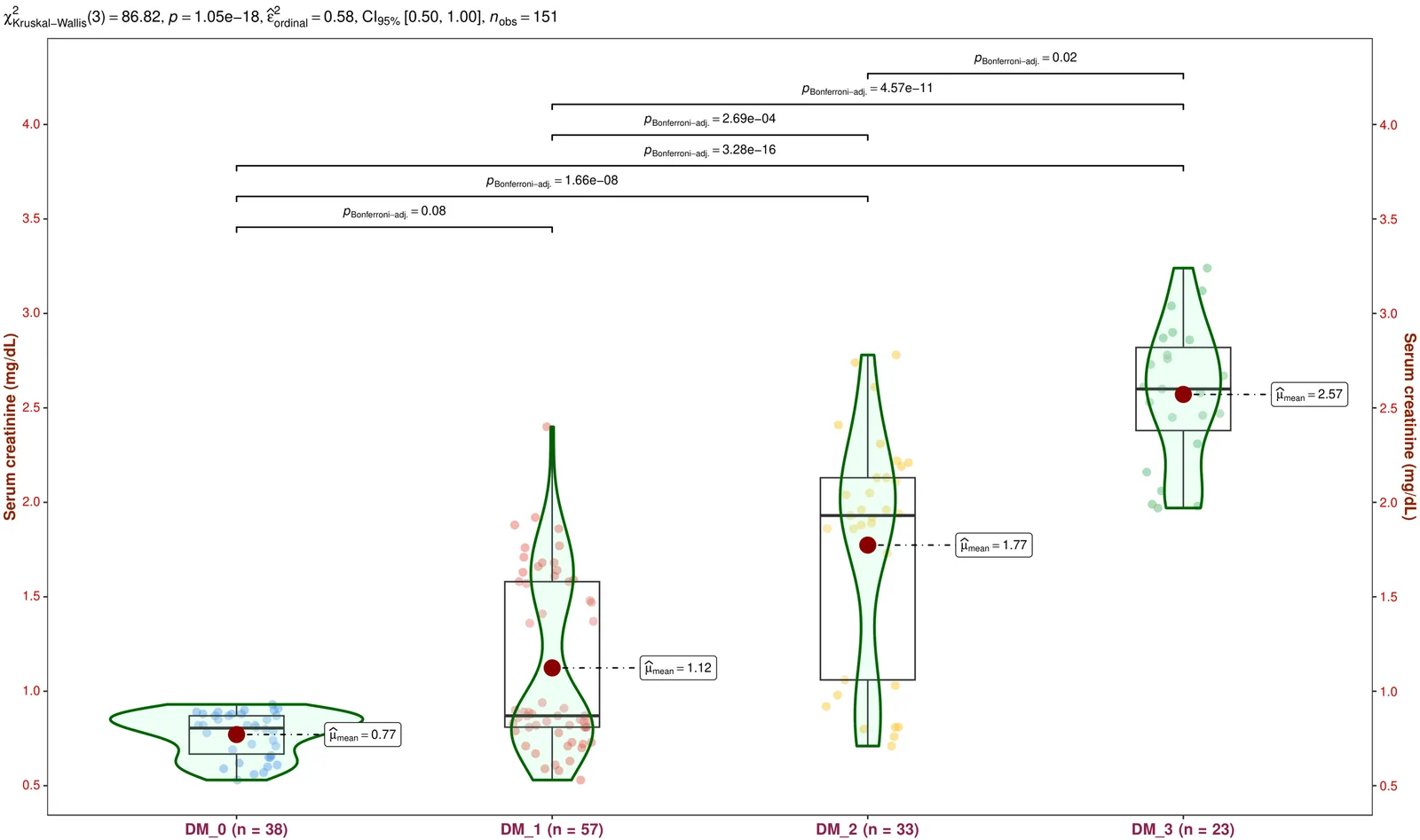
Serum creatinine levels of the study participants. The violin, jitter, and box-and-whisker plots illustrate the serum creatinine levels of the study participants (n = 151). The red dots denote the mean values. The Kruskal-Wallis test was used to assess these data. For post hoc analysis, we used the Bonferroni test. A p-value < 0.05 was considered statistically significant. The digits (i.e., 0, 1, 2, and 3) in the groups shown on the x-axis represent the number of microvascular complications. T2DM: type 2 diabetes mellitus R software (version 4.6.2) was used to generate this plot [24].

Glycemic parameters

Figure 5 illustrates the FBS levels of the participants. The median FBS level was 189.0 (163.5-217.0) mg/dL. The median FBS levels among participants with 0, 1, 2, and 3 complications were 167.0 (144.3-193.5), 175.0 (158.0-198.0), 210.0 (181.0-228.0), and 227.0 (210.0-248.5) mg/dL, respectively (p < 0.001). The mean FBS levels showed an increasing trend with the number of complications. The participants’ FBS levels are also shown in Table 2.

**Figure 5:**
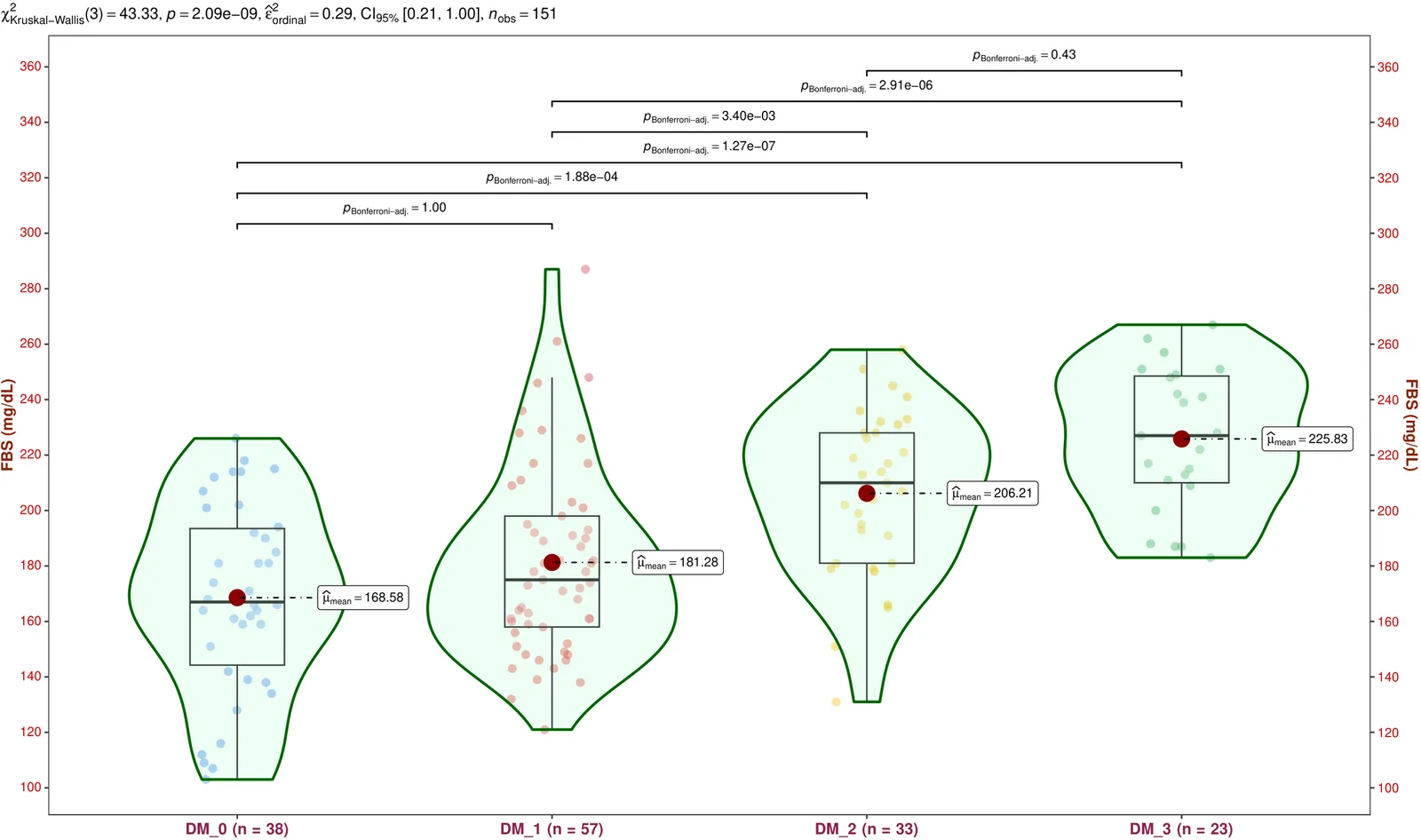
FBS levels of the study participants. The violin, jitter, and box-and-whisker plots illustrate the FBS levels of the study participants (n = 151). The red dots denote the mean values. The Kruskal-Wallis test was used to assess these data. For post hoc analysis, we used the Bonferroni test. A p-value < 0.05 was considered statistically significant. The digits (i.e., 0, 1, 2, and 3) in the groups shown on the x-axis represent the number of microvascular complications. FBS: fasting blood sugar; T2DM: type 2 diabetes mellitus R software (version 4.6.2) was used to generate this plot [24].

**Table 2:** Glycemic parameters of the study participants. Continuous data are expressed as medians and IQRs. These data were analyzed using the Kruskal-Wallis test, and H-statistics were obtained. A p-value < 0.05 was considered statistically significant. FBS: fasting blood sugar; HbA1c: glycated hemoglobin

Parameters	Total participants (n = 151)	Participants without any complication (n = 38)	Participants with one complication (n = 57)	Participants with two complications (n = 33)	Participants with three complications (n = 23)	Test statistics	p-value
FBS (mg/dL)	189.0 (163.5-217.0)	167.0 (144.3-193.5)	175.0 (158.0-198.0)	210.0 (181.0-228.0)	227.0 (210.0-248.5)	43.33	<0.001
HbA1c (%)	7.31 (7.03-7.89)	7.31 (6.99-7.82)	7.12 (6.90-7.35)	7.62 (7.24-8.01)	7.87 (7.31-8.54)	27.23	<0.001
Serum insulin (µIU/mL)	4.91 (4.04-6.03)	5.74 (5.00-7.11)	4.18 (3.78-4.77)	5.17 (4.26-6.16)	5.61 (4.59-6.77)	33.52	<0.001

Figure 6 illustrates the HbA1c levels of the participants. The median HbA1c level was 7.31 (7.03-7.89%). The median HbA1c levels among participants with 0, 1, 2, and 3 complications were 7.31 (6.99-7.82%), 7.12 (6.90-7.35%), 7.62 (7.24-8.01%), and 7.87 (7.31-8.54%), respectively (p < 0.001). The differences in HbA1c levels between participants with 0-1 and 2-3 complications were statistically significant. The participants’ HbA1c levels are also shown in Table 2.

**Figure 6:**
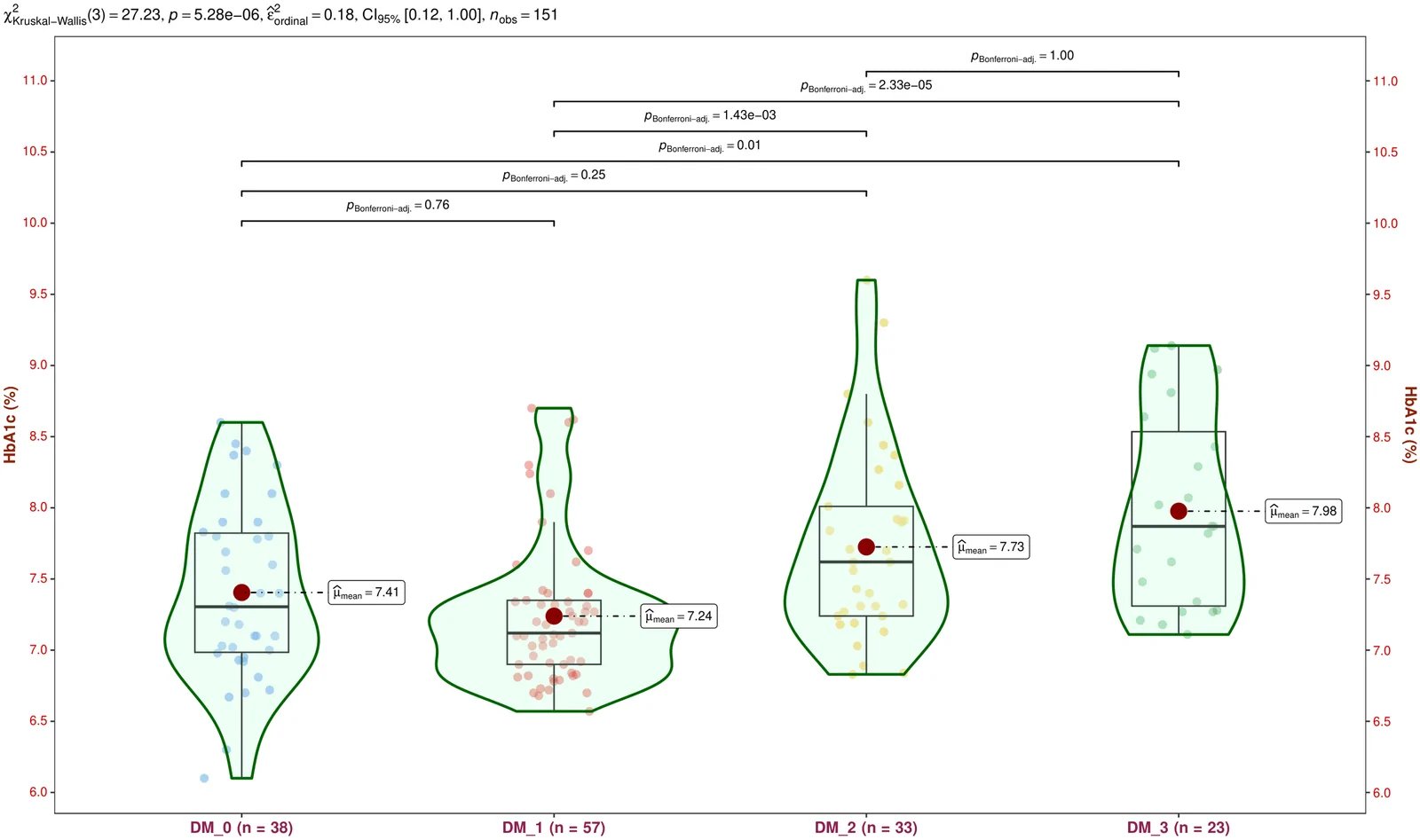
HbA1c levels of the study participants. The violin, jitter, and box-and-whisker plots illustrate the HbA1c levels of the study participants (n = 151). The red dots denote the mean values. The Kruskal-Wallis test was used to assess these data. For post hoc analysis, we used the Bonferroni test. A p-value < 0.05 was considered statistically significant. The digits (i.e., 0, 1, 2, and 3) in the groups shown on the x-axis represent the number of microvascular complications. HbA1c: glycated hemoglobin; T2DM: type 2 diabetes mellitus R software (version 4.6.2) was used to generate this plot [24].

Figure 7 illustrates the serum insulin levels of the participants. The median serum insulin level was 4.91 (4.04-6.03) µIU/mL. The median serum insulin levels among participants with 0, 1, 2, and 3 complications were 5.74 (5.00-7.11), 4.18 (3.78-4.77), 5.17 (4.26-6.16), and 5.61 (4.59-6.77) µIU/mL, respectively (p < 0.001). The mean serum insulin level among participants with one complication was lower than that among the other participants. The differences in median serum insulin levels between participants with one complication and the other groups were statistically significant. The participants’ serum insulin levels are also shown in Table 2.

**Figure 7:**
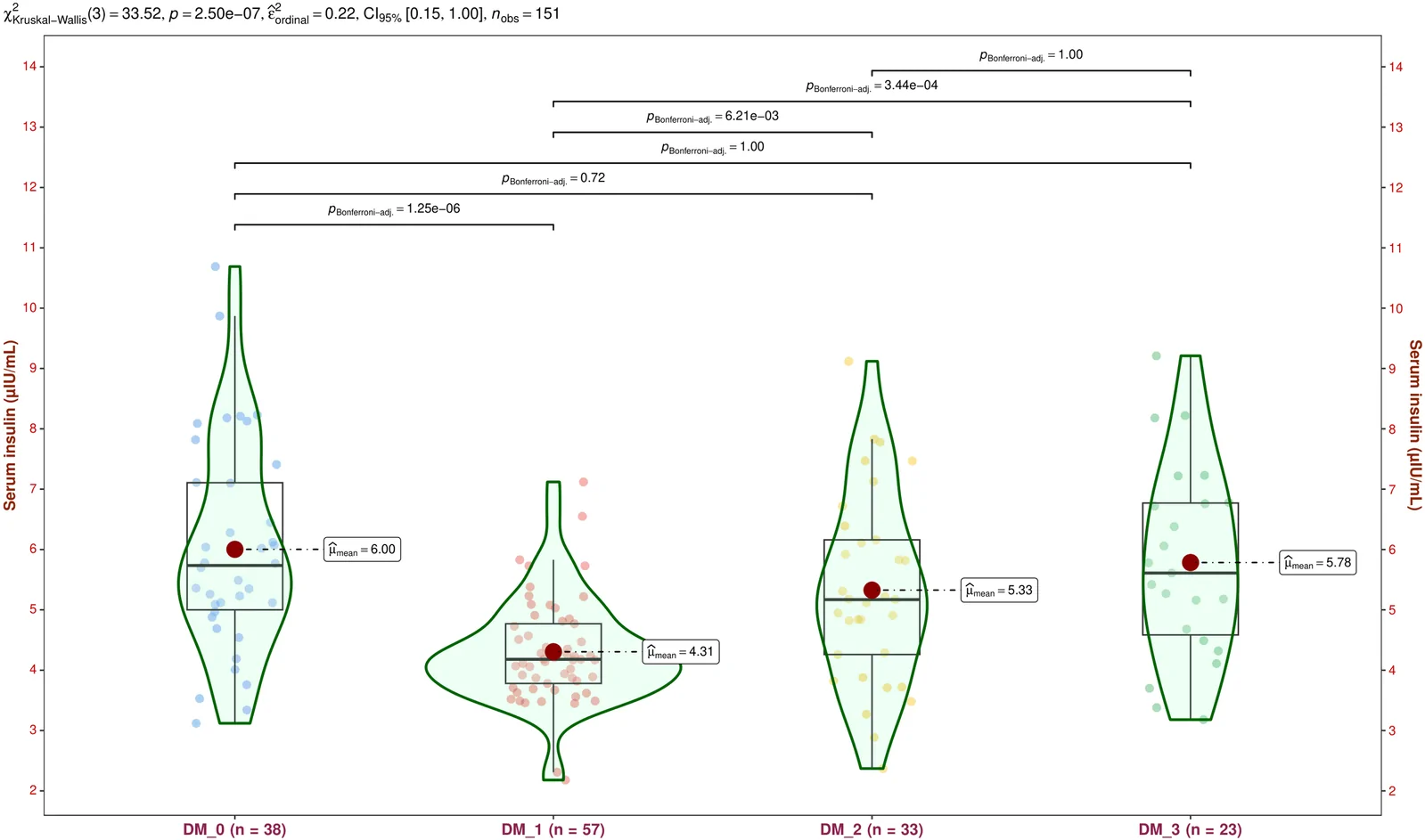
Serum insulin levels of the study participants. The violin, jitter, and box-and-whisker plots illustrate the serum insulin levels of the study participants (n = 151). The red dots denote the mean values. The Kruskal-Wallis test was used to assess these data. For post hoc analysis, we used the Bonferroni test. A p-value < 0.05 was considered statistically significant. The digits (i.e., 0, 1, 2, and 3) in the groups shown on the x-axis represent the number of microvascular complications. T2DM: type 2 diabetes mellitus R software (version 4.6.2) was used to generate this plot [24].

Lipid parameters

Figure 8 illustrates the serum total cholesterol levels of the participants. The median serum total cholesterol level was 178.0 (153.0-203.0) mg/dL. The median serum total cholesterol levels among participants with 0, 1, 2, and 3 complications were 161.5 (145.5-189.0), 173.0 (146.0-189.0), 197.0 (164.0-211.0), and 209.0 (186.5-218.5) mg/dL, respectively (p < 0.001). The mean serum total cholesterol levels increased with the number of complications. The participants’ serum total cholesterol levels are also shown in Table 3.

**Figure 8:**
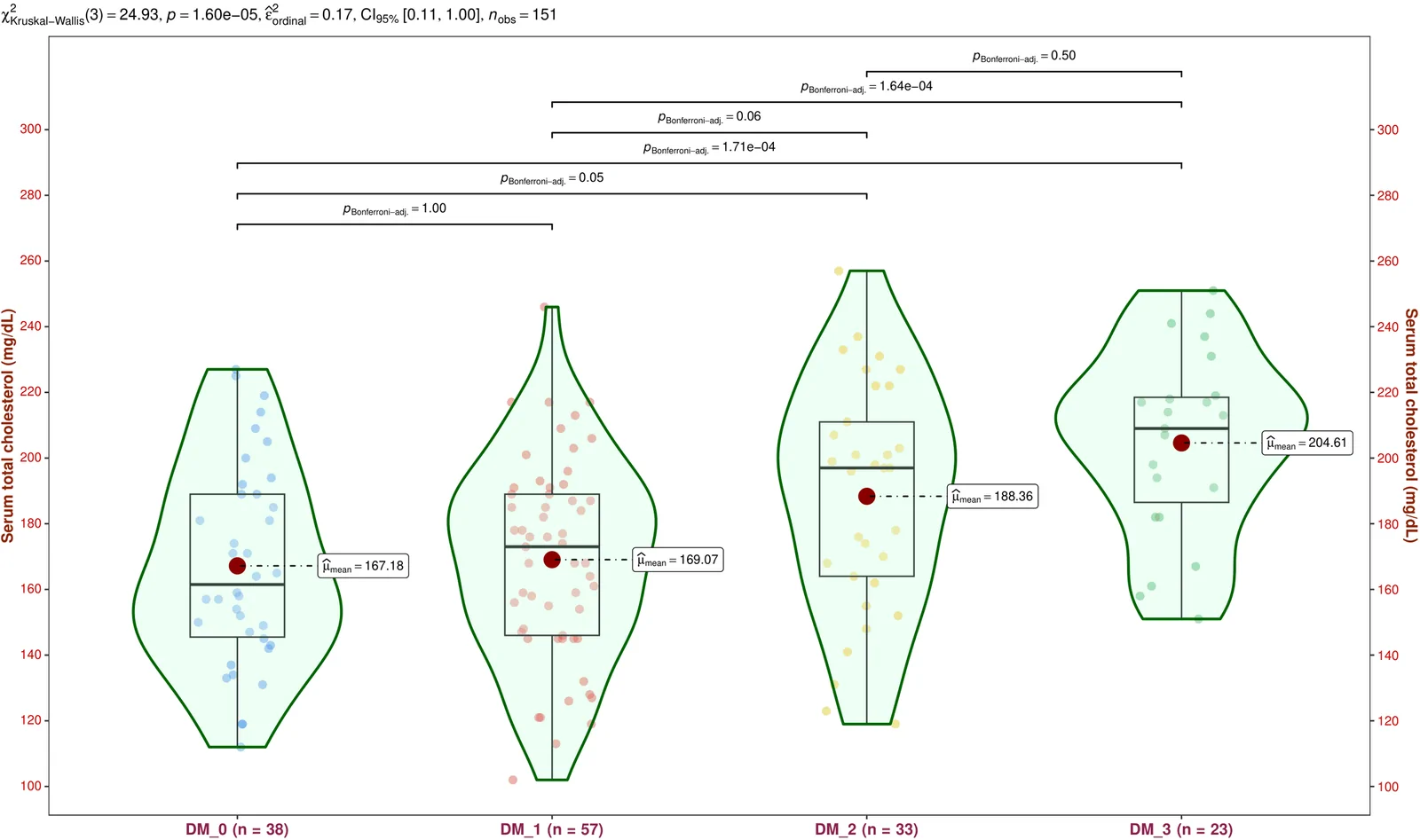
Serum total cholesterol levels of the study participants. The violin, jitter, and box-and-whisker plots illustrate the serum total cholesterol levels of the study participants (n = 151). The red dots denote the mean values. The Kruskal-Wallis test was used to assess these data. For post hoc analysis, we used the Bonferroni test. A p-value < 0.05 was considered statistically significant. The digits (i.e., 0, 1, 2, and 3) in the groups shown on the x-axis represent the number of microvascular complications. T2DM: type 2 diabetes mellitus R software (version 4.6.2) was used to generate this plot [24].

**Table 3:** Lipid parameters of the study participants. Continuous data are expressed as medians and IQRs. These data were analyzed using the Kruskal-Wallis test, and H-statistics were obtained. A p-value < 0.05 was considered statistically significant. HDL: high-density lipoprotein; LDL: low-density lipoprotein; VLDL: very-low-density lipoprotein

Parameters	Total participants (n = 151)	Participants without any complication (n = 38)	Participants with one complication (n = 57)	Participants with two complications (n = 33)	Participants with three complications (n = 23)	Test statistics	p-value
Serum total cholesterol (mg/dL)	178.0 (153.0-203.0)	161.5 (145.5-189.0)	173.0 (146.0-189.0)	197.0 (164.0-211.0)	209.0 (186.5-218.5)	24.93	<0.001
Serum triglyceride (mg/dL)	136.0 (116.0-161.5)	119.5 (106.0-134.8)	132.0 (116.0-147.0)	152.0 (134.0-168.0)	162.0 (142.5-180.5)	30.99	<0.001
Serum HDL (mg/dL)	38.0 (35.0-41.0)	38.5 (35.3-40.8)	38.0 (36.0-41.0)	39.0 (36.0-42.0)	35.0 (30.0-38.5)	9.17	0.027
Serum LDL (mg/dL)	109.0 (82.5-123.5)	91.5 (77.3-118.8)	103.0 (81.0-116.0)	118.0 (93.0-127.0)	125.0 (117.0-132.0)	23.44	<0.001
Serum VLDL (mg/dL)	23.0 (18.5-30.0)	24.5 (17.3-30.0)	21.0 (16.0-27.0)	24.0 (23.0-30.0)	23.0 (19.5-30.5)	5.56	0.135
Cholesterol-HDL ratio	4.59 (3.97-5.51)	4.34 (3.71-5.14)	4.38 (3.96-4.97)	4.83 (4.11-5.64)	6.08 (5.40-6.40)	30.78	<0.001

Figure 9 illustrates the serum triglyceride levels of the participants. The median serum triglyceride level was 136.0 (116.0-161.5) mg/dL. The median serum triglyceride levels among participants with 0, 1, 2, and 3 complications were 119.5 (106.0-134.8), 132.0 (116.0-147.0), 152.0 (134.0-168.0), and 162.0 (142.5-180.5) mg/dL, respectively (p < 0.001). The mean serum triglyceride levels increased with the number of complications. The differences in serum triglyceride levels between participants with 0-1 and 2-3 complications were statistically significant. The participants’ serum triglyceride levels are also shown in Table 3.

**Figure 9:**
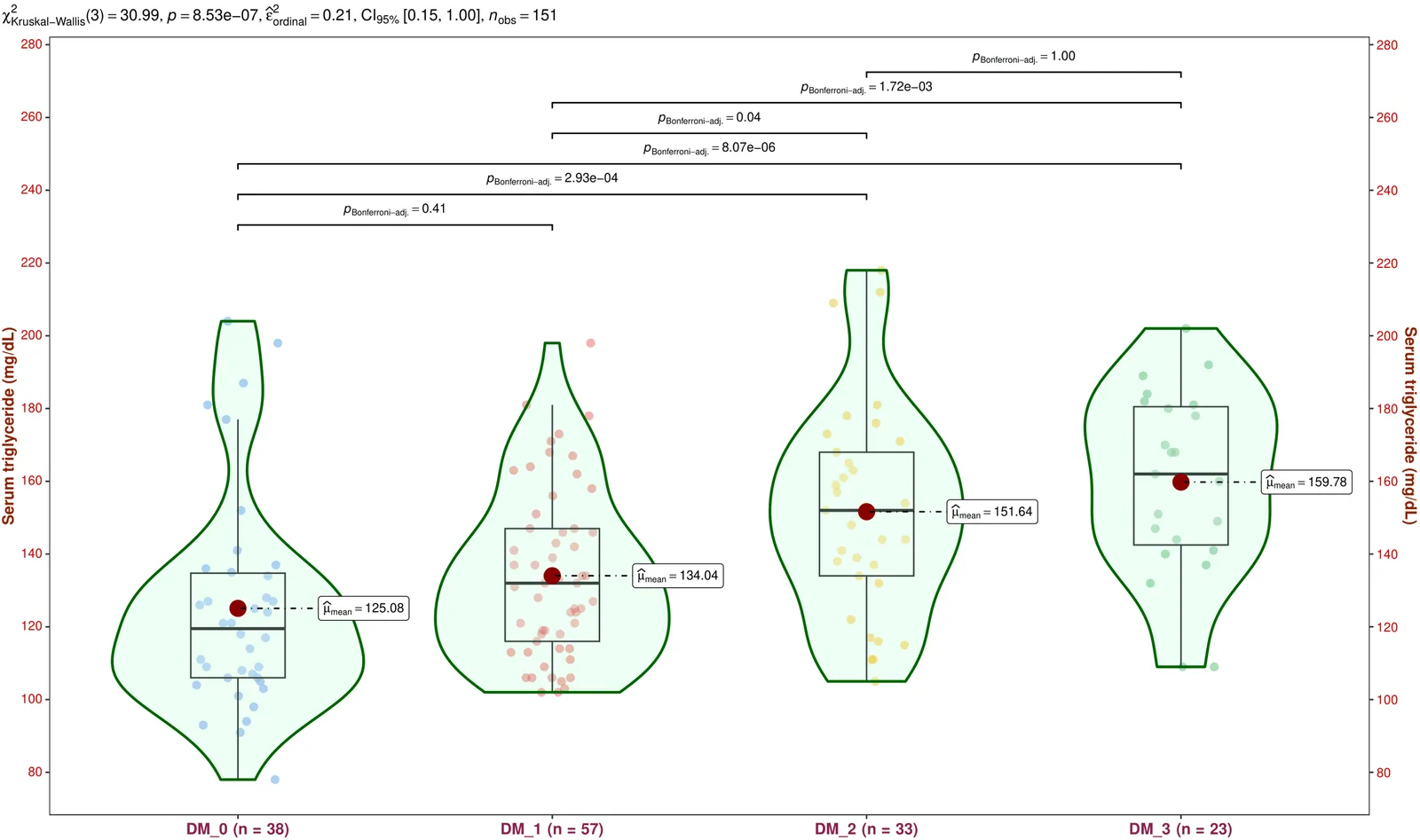
Serum triglyceride levels of the study participants. The violin, jitter, and box-and-whisker plots illustrate the serum triglyceride levels of the study participants (n = 151). The red dots denote the mean values. The Kruskal-Wallis test was used to assess these data. For post hoc analysis, we used the Bonferroni test. A p-value < 0.05 was considered statistically significant. The digits (i.e., 0, 1, 2, and 3) in the groups shown on the x-axis represent the number of microvascular complications. T2DM: type 2 diabetes mellitus R software (version 4.6.2) was used to generate this plot [24].

sRAGE, HOMA-IR, and TyG index

Figure 10 illustrates the sRAGE levels of the participants. The median sRAGE level was 276.4 (201.8-413.9) pg/mL. The median sRAGE levels among participants with 0, 1, 2, and 3 complications were 410.2 (318.6-551.4), 281.3 (226.4-410.4), 226.4 (167.3-328.5), and 143.4 (106.7-178.8) pg/mL, respectively (p < 0.001). The mean sRAGE levels showed a decreasing trend with increasing numbers of complications. Post hoc analyses using the Bonferroni test revealed statistically significant differences among the groups, except between participants with one and two complications. The participants’ sRAGE levels are also shown in Table 4.

**Figure 10:**
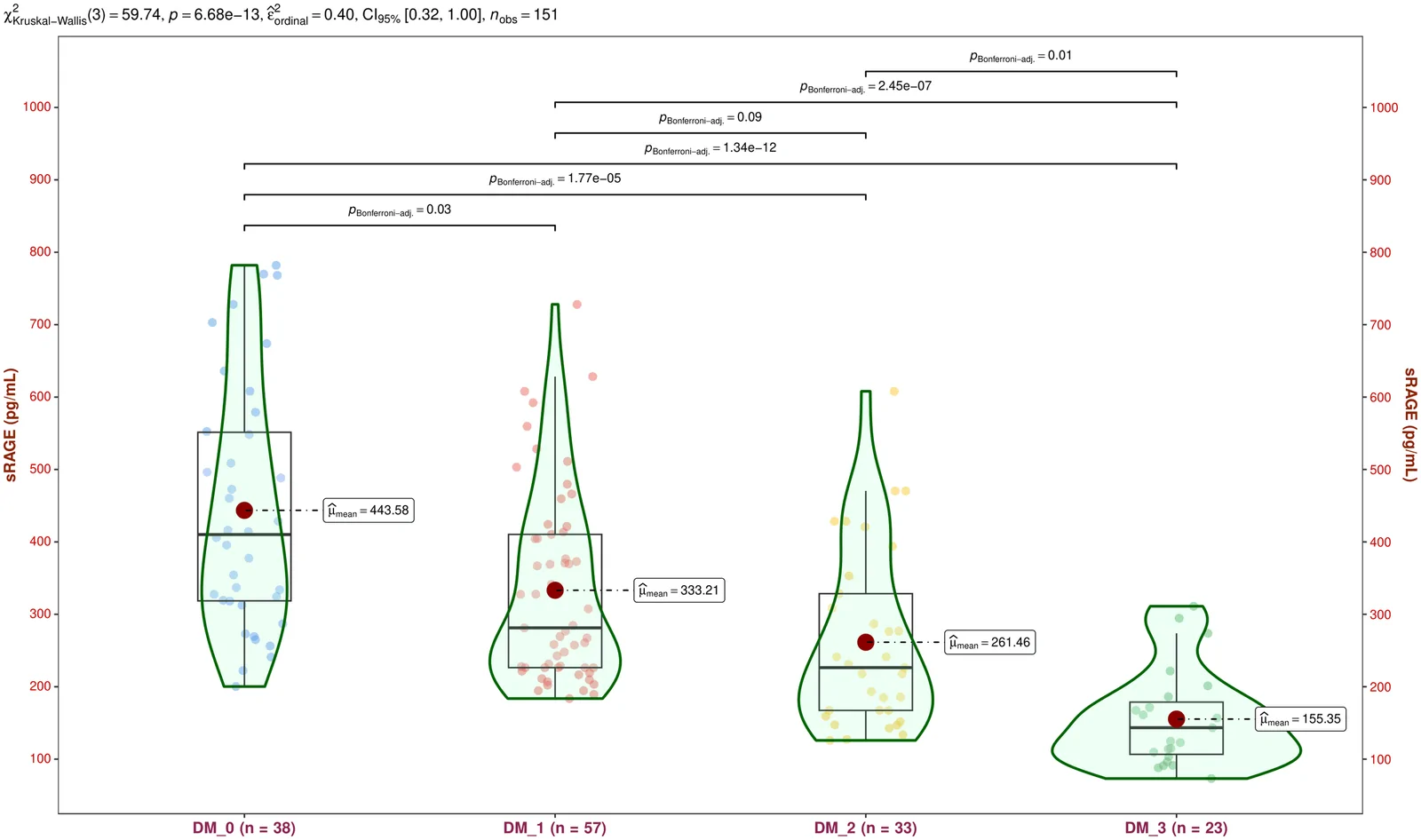
sRAGE levels of the study participants. The violin, jitter, and box-and-whisker plots illustrate the sRAGE levels of the study participants (n = 151). The red dots denote the mean values. The Kruskal-Wallis test was used to assess these data. For post hoc analysis, we used the Bonferroni test. A p-value < 0.05 was considered statistically significant. The digits (i.e., 0, 1, 2, and 3) in the groups shown on the x-axis represent the number of microvascular complications. sRAGE: soluble receptor for advanced glycation end-products; T2DM: type 2 diabetes mellitus R software (version 4.6.2) was used to generate this plot [24].

**Table 4:** sRAGE, HOMA-IR, and TyG index of the study participants. Continuous data are expressed as medians and IQRs. These data were analyzed using the Kruskal-Wallis test, and H-statistics were obtained. A p-value < 0.05 was considered statistically significant. HOMA-IR: homeostatic model assessment of insulin resistance; sRAGE: soluble receptor for advanced glycation end-products; TyG: triglyceride-glucose index

Parameters	Total participants (n = 151)	Participants without any complication (n = 38)	Participants with one complication (n = 57)	Participants with two complications (n = 33)	Participants with three complications (n = 23)	Test statistics	p-value
sRAGE (pg/mL)	276.4 (201.8-413.9)	410.2 (318.6-551.4)	281.3 (226.4-410.4)	226.4 (167.3-328.5)	143.4 (106.7-178.8)	59.74	<0.001
HOMA-IR	2.18 (1.78-3.03)	2.19 (1.92-2.88)	1.86 (1.57-2.11)	2.71 (1.98-3.45)	3.14 (2.42-3.90)	37.26	<0.001
TyG index	9.44 (9.22-9.71)	9.20 (8.90-9.38)	9.35 (9.22-9.54)	9.67 (9.50-9.79)	9.80 (9.62-9.97)	50.54	<0.001

Figure 11 illustrates the HOMA-IR values of the participants. The median HOMA-IR value was 2.18 (1.78-3.03). The median HOMA-IR values among participants with 0, 1, 2, and 3 complications were 2.19 (1.92-2.88), 1.86 (1.57-2.11), 2.71 (1.98-3.45), and 3.14 (2.42-3.90), respectively (p < 0.001). The participants’ HOMA-IR values are also shown in Table 4.

**Figure 11:**
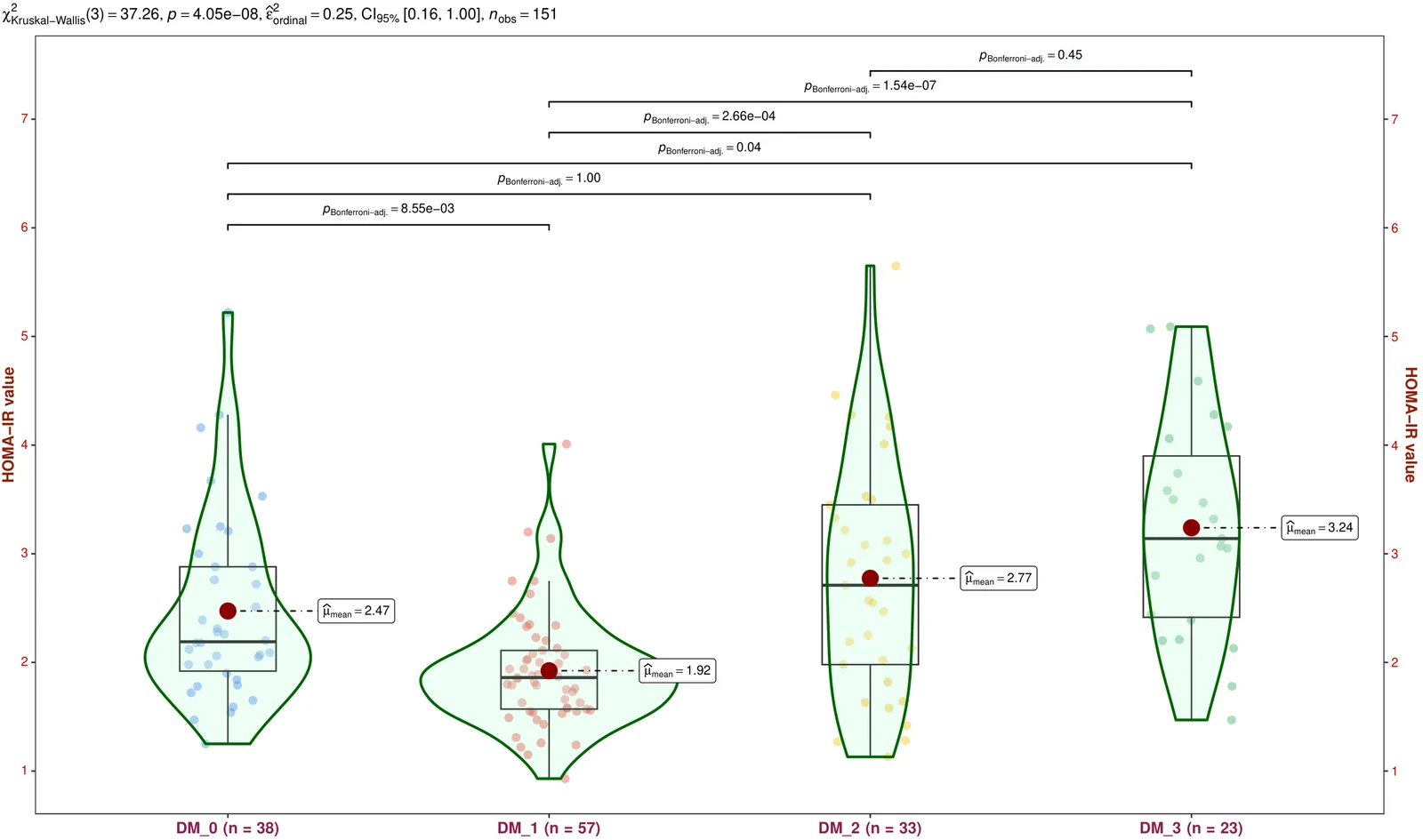
HOMA-IR values of the study participants. The violin, jitter, and box-and-whisker plots illustrate the HOMA-IR values of the study participants (n = 151). The red dots denote the mean values. The Kruskal-Wallis test was used to assess these data. For post hoc analysis, we used the Bonferroni test. A p-value < 0.05 was considered statistically significant. The digits (i.e., 0, 1, 2, and 3) in the groups shown on the x-axis represent the number of microvascular complications. HOMA-IR: homeostatic model assessment of insulin resistance; T2DM: type 2 diabetes mellitus R software (version 4.6.2) was used to generate this plot [24].

Figure 12 illustrates the TyG index values of the participants. The median TyG index was 9.44 (9.22-9.71). The median TyG index values among participants with 0, 1, 2, and 3 complications were 9.20 (8.90-9.38), 9.35 (9.22-9.54), 9.67 (9.50-9.79), and 9.80 (9.62-9.97), respectively (p < 0.001). The mean TyG index values showed an increasing trend with the number of complications. The differences in TyG index values between participants with 0-1 and 2-3 complications were statistically significant. The participants’ TyG index values are also shown in Table 4.

**Figure 12:**
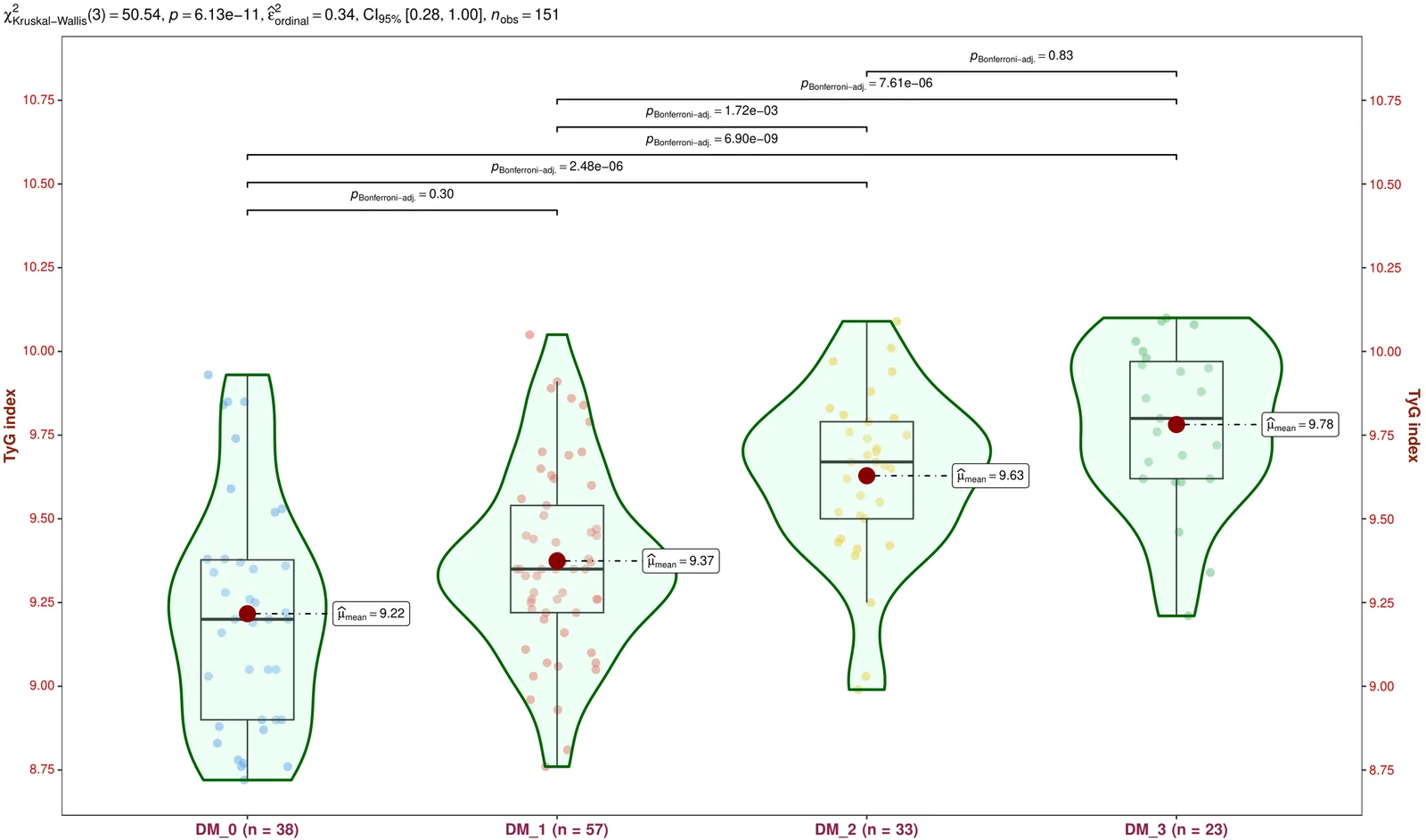
TyG index values of the study participants. The violin, jitter, and box-and-whisker plots illustrate the TyG index values of the study participants (n = 151). The red dots denote the mean values. The Kruskal-Wallis test was used to assess these data. For post hoc analysis, we used the Bonferroni test. A p-value < 0.05 was considered statistically significant. The digits (i.e., 0, 1, 2, and 3) in the groups shown on the x-axis represent the number of microvascular complications. T2DM: type 2 diabetes mellitus; TyG index: triglyceride-glucose index R software (version 4.6.2) was used to generate this plot [24].

Correlation of various parameters

Figure 13 demonstrates the correlation coefficients among various parameters. We observed positive correlations between FBS and the TyG index (r = 0.833, 95% CI = 0.776 to 0.878, p < 0.001), serum triglycerides and the TyG index (r = 0.827, 95% CI = 0.769 to 0.872, p < 0.001), serum insulin and HOMA-IR (r = 0.819, 95% CI = 0.758 to 0.865, p < 0.001), age and duration of T2DM (r = 0.817, 95% CI = 0.756 to 0.864, p < 0.001), FBS and HOMA-IR (r = 0.731, 95% CI = 0.646 to 0.797, p < 0.001), and serum total cholesterol and serum triglycerides (r = 0.715, 95% CI = 0.627 to 0.785, p < 0.001). We observed negative correlations between sRAGE and serum creatinine (r = -0.561, 95% CI = -0.661 to -0.440, p < 0.001), the TyG index (r = -0.332, 95% CI = -0.467 to -0.181, p < 0.001), duration of T2DM (r = -0.329, 95% CI = -0.465 to -0.179, p < 0.001), and age (r = -0.312, 95% CI = -0.449 to -0.160, p < 0.001). There was a weak positive correlation between sRAGE and serum insulin (r = 0.220, 95% CI = 0.062 to 0.367, p = 0.007). The correlation between sRAGE and HOMA-IR (r = -0.002, 95% CI = -0.162 to 0.157, p = 0.976) was not statistically significant.

**Figure 13:**
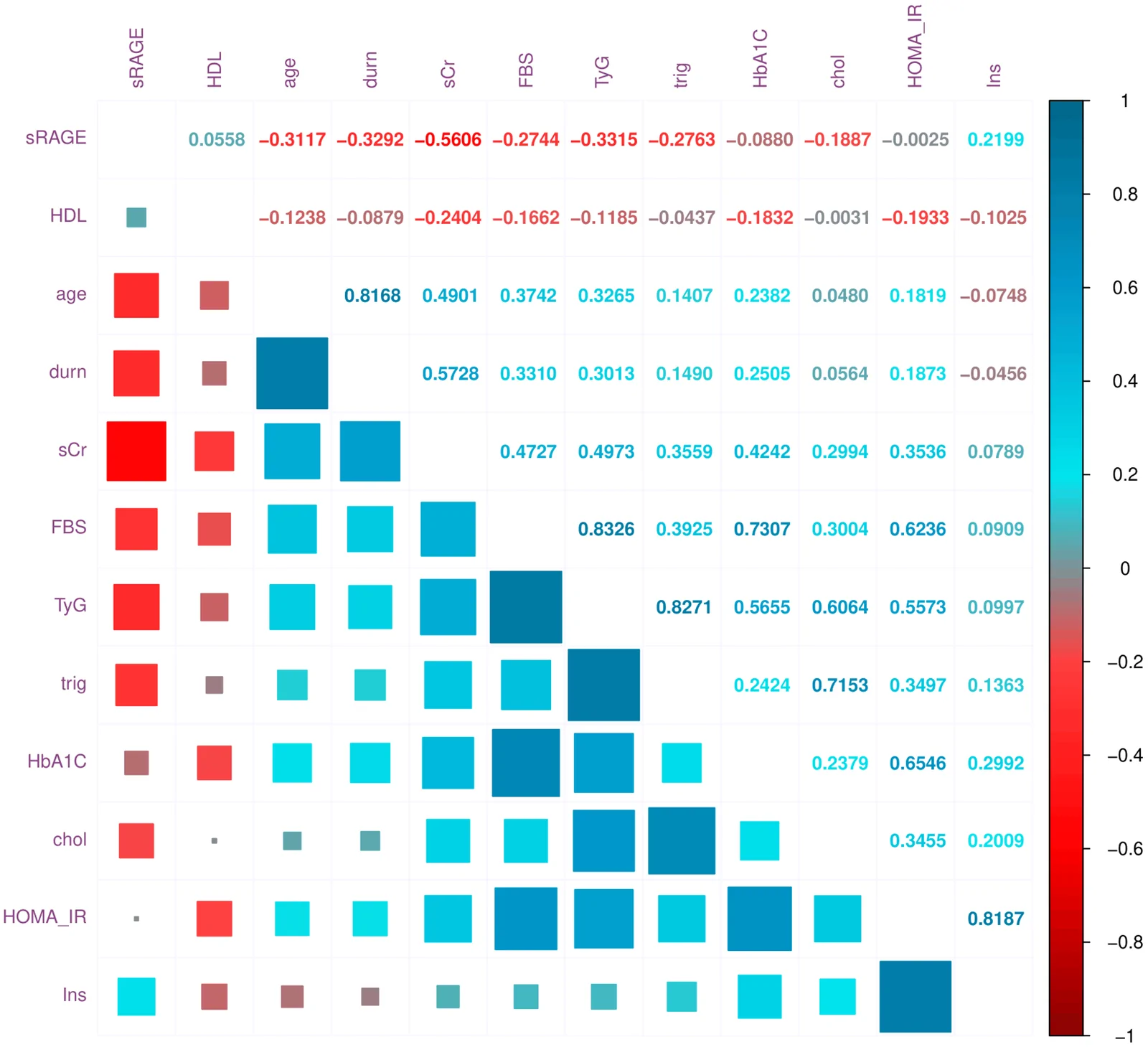
Correlation of various parameters. The correlation plot shows the degree of association among the parameters. The correlation coefficients range from +1 (strong positive correlation) to -1 (strong negative correlation). Chol: serum total cholesterol; durn: duration of T2DM; FBS: fasting blood sugar; HbA1c: glycated hemoglobin; HDL: high-density lipoprotein; HOMA_IR: homeostatic model assessment of insulin resistance; Ins: serum insulin; sCr: serum creatinine; sRAGE: soluble receptor for advanced glycation end-products; T2DM: type 2 diabetes mellitus; trig: serum triglycerides; TyG: triglyceride-glucose index R software (version 4.6.2) was used to generate this plot [24].

## Discussion

To the best of our knowledge, this is the first study to assess serum sRAGE levels, fasting serum insulin concentrations, HOMA-IR, and the TyG index in diabetic patients with and without microvascular complications. Interestingly, our study revealed that serum insulin and sRAGE levels were lower in participants without any complications than in those with two to three complications. The highest values of HOMA-IR and the TyG index were observed among participants with all three microvascular complications. We also observed the highest values for the following parameters among participants with all three microvascular complications: age, duration of T2DM, serum creatinine, FBS, HbA1c, serum total cholesterol, and serum triglycerides.

The risk of diabetic microvascular complications increases with advancing age and duration of T2DM [2,4,7]. This risk is also influenced by glycemic control, lifestyle modifications, dietary habits, stress levels, sleep quality, medication adherence, comorbidities, and genetics [2-6]. Renal function deteriorates with increasing duration of T2DM and sustained hyperglycemia [4,25]. In our study, declining renal function was evidenced by reduced eGFR and elevated serum creatinine levels among participants with more microvascular complications. However, the bimodal distribution of serum creatinine levels among participants with one to two complications could be explained by the fact that not all participants in these two groups had diabetic nephropathy.

FBS levels showed a trend with the number of complications. However, serum insulin and HbA1c values did not clearly exhibit either an increasing or decreasing trend across the study groups. These variations could be explained by patient compliance, lifestyle modifications, dietary habits, comorbidities, and genetics [2,7,18,20]. Dysglycemia, dietary patterns, and genetics often affect an individual’s overall lipid profile [19,26]. Restricting saturated fatty acids and emphasizing polyunsaturated and monounsaturated fatty acids could result in favorable glycemic and lipid profiles [26]. At the polygenic level, dietary quality might differentiate the risk of dyslipidemia and atherosclerosis in genetically susceptible individuals [26]. Our study population demonstrated an increasing trend in serum total cholesterol and serum triglyceride levels with increasing numbers of microvascular complications.

Although average sRAGE levels declined across the study groups, sRAGE levels varied within the study population. These variations might be attributable to genetic polymorphisms. Various single-nucleotide polymorphisms (SNPs) in the AGER gene have been investigated in patients with T2DM. Important SNPs include rs2070600, rs1800624, rs1800625, rs184003, and rs55640627 [27,28]. The rs2070600 SNP in the AGER gene has been associated with reduced RAGE proteolysis. Hence, this SNP has been linked to decreased sRAGE levels in patients with T2DM [29,30]. Of the aforementioned SNPs, rs2070600 and rs1800624 are correlated with sRAGE levels [29]. Particular interest has focused on the rs2070600 G/A SNP in exon 3, which causes a glycine (G)-to-serine (S) substitution at position 82 of the amino acid chain because it is situated in the ligand-binding site and could significantly affect the AGE/RAGE interaction [30]. There was a strong negative correlation between the CA, TA, and TT haplotypes (alleles of rs2070600 and rs1800624) and sRAGE levels compared with the CT haplotype [29].

Increased AGE synthesis due to chronic hyperglycemia might reduce sRAGE levels because sRAGE interferes with AGE-RAGE interactions [10-12,15]. As the clearance rate of the sRAGE-AGE complex increases and circulating free sRAGE levels decrease, overall sRAGE concentrations might also decrease [15]. This might explain the reduced sRAGE levels across our study groups. Another RAGE ligand, S100A12, is upregulated in diabetic individuals with insulin resistance, which might also lead to reduced sRAGE levels in patients with chronic diabetes [15]. However, our study did not identify any significant correlations between sRAGE and HOMA-IR values.

Overweight and obese individuals generally have insulin resistance and dyslipidemia compared with normal-weight individuals [19,23]. Increased serum triglyceride levels in individuals with greater visceral adiposity may be attributable to insulin resistance [23]. These findings support the plausibility of serum triglycerides as a surrogate biomarker for insulin resistance. The TyG index can be used in resource-limited settings [23]. In our study, we did not find strong correlations among the TyG index, HOMA-IR, and sRAGE levels. We are currently assessing the association between AGER gene SNPs and sRAGE levels among our study participants.

Strengths and limitations

The strengths of this study included the analyses of serum sRAGE levels, fasting serum insulin, HOMA-IR, and the TyG index in diabetic patients with and without microvascular complications, as well as the correlations among these parameters. This study could have been improved in several ways. First, we could have evaluated and compared these values at multiple assessment time points to examine their trends over time. Second, we did not categorize participants by complication type. Such categorization would have provided a clearer picture of the association between sRAGE levels and different microvascular complications. Third, we could not evaluate the roles of concomitant medications and comorbidity severity because of the study design. Fourth, the sample size could not be increased because of financial constraints. A larger sample size would have helped us better understand the variability in sRAGE levels and their associations with SNPs.

## Conclusions

In our study, we found that sRAGE levels decreased with increasing numbers of microvascular complications. The TyG index and HOMA-IR were higher among participants with two to three complications. We did not find strong correlations between sRAGE levels and any of the studied parameters. Our ongoing analysis of SNPs might help us understand the variability in sRAGE levels across the study population. We recommend prospective studies with longer follow-up periods and larger sample sizes to determine long-term changes in sRAGE levels and their predictive value for insulin resistance among diabetic patients with and without microvascular complications.
